# Crafting the success and failure of decentralized marine management

**DOI:** 10.1007/s13280-022-01763-7

**Published:** 2022-07-25

**Authors:** Jean Wencélius, Matthew Lauer, Tamatoa Bambridge

**Affiliations:** 1 Department of Anthropology, San Diego State University, 5500 Campanile Dr., San Diego, CA 92182-6040 USA; 2 PSL Paris University: EPHE-UPVD-CNRS, USR 3278 CRIOBE, BP 1013 Papetoai, 98729 Moorea, French Polynesia

**Keywords:** Community-based marine resource management, Expert/non-expert knowledge, French Polynesia, Marine conservation, Rāhui, Reef fisheries

## Abstract

This paper presents an ethnographic case study of the design and revision of a decentralized marine management scheme implemented on the island of Moorea, French Polynesia named *Plan de Gestion de l’Espace Maritime* (PGEM). Drawing on an analysis of over 50 consultative workshops and meetings, held from 2018 to 2021 during the PGEM revision, we document the materials, discourses, and practices local stakeholders (e.g., fishers, cultural and environmental activists, government staff, and scientists) combine to build their interpretations of PGEM success or failure. We examine the diversity of domains these interpretations draw from (ecology, marine livelihoods, culture, religion, and politics) and how they are put into practice in people’s engagement with—or resistance to—the local marine management and governance design. Our results highlight how the controversies around the revision of Moorea’s PGEM overflowed the boundaries of ecology as construed by scientific experts. Stakeholders interpreted “marine resource management” as something well beyond just “marine resources” to include politics, identity, Polynesian cosmology, and livelihoods. Our findings provide generalizable patterns for understanding how natural-resource management policies are received and repurposed by local actors.

**Supplementary Information:**

The online version contains supplementary material available at 10.1007/s13280-022-01763-7.

## Introduction

Empowering local communities and forwarding their stewardship over natural resources have become, over the past decades, the dominant paradigm of conservation and sustainable development agendas across the globe (Berkes 2004), and more particularly in the Pacific. The lessons provided by the limited success of state-led, top-down efforts to ban or restrict human activities in designated areas (Bennett and Dearden 2014) as well as the rich body of academic work describing customary forms of management (e.g., Johannes 1978; Berkes 1989; Bambridge 2016; Lauer 2017) have given momentum to the idea of devolving resource management to local communities.

Since the 1980s, Oceania has been at the forefront in the implementation of various initiatives of marine co-management or Community-Based Marine Resource Management (CBMRM; Govan 2009). Through the fast-growing number of Locally Managed Marine Areas (LMMA) or enabling legislation guaranteeing customary marine tenure, the Pacific has witnessed a ‘rennaissance’ of locally based and community-driven initiatives of environmental and resource management (Johannes 2002; Govan 2009; Friedlander 2018) making CBMRM the primary regional strategy and policy orientation across the Pacific (Cinner et al. 2012; Cohen et al. 2015; Hanich et al. 2018). Regional organizations, conservation NGOs, and private foundations now stress the importance for state agencies to support and upscale CBMRM initiatives (SPC 2010, 2021; Karcher et al. 2020; Steenbergen et al. 2022). Yet, the process by which these seemingly simple strategies move from bullet points in project plan documents towards gaining reality at the project site are not well understood. In this paper, we examine ethnographically the design and revision of a network of coral-reef protected areas established on the island of Moorea in French Polynesia (FP) as a decentralized marine spatial planning and resource management regime known as the *Plan de Gestion de l’Espace Maritime* (PGEM, Marine Spatial Management Scheme).

Our main line of enquiry concerns the assessment of CBMRM and how “success” or “failure” is constructed in the everyday practices of those who participate in or who are affected by management schemes. The gold standard of “success” is built upon peer-reviewed scientific literature compiled by trained experts reporting discrete ecological metrics about the change in fish biomass or the quality of marine habitats within the boundaries of no-take zones (Thiault et al. 2019). Socio-cultural indicators may also be used—such as the degree of engagement of stakeholders—to evaluate the successful (or unsuccessful) outcomes of marine resource management (Maliao et al. 2009). Both types of assessments share two common elements. First, success or failure is composed by experts construing the outcomes of CBMRM projects as an inherent set of attributes that can be rationally assessed from an external position (Giakoumi et al. 2018). Second, CBMRM is approached as an abstract model that can be copied from one context to the next (Jupiter et al. 2014). When it fails, it is assumed that the tool either malfunctioned or lacked some key elements.

Here, we approach CBMRM not as a self-evident abstraction, but as a process of composition carried out through the active practical work of not only conservation managers but other local actors such as fishers or activists. In some ways, our approach aligns with a growing body of literature—to which we have contributed (Hunter et al. 2018; Fabre et al. 2021)—documenting the local perceptions and conceptions communities have of conservation efforts affecting their everyday lives (Bennett and Dearden 2014). But here we build on another body of literature, mostly in development studies (Mosse 2005), and we ask not whether Moorea’s PGEM failed or not, but *how success or failure is produced* through the discourses and practices of stakeholders engaged in Moorea’s local marine governance and management.

Importantly, the coherence of complex socio-ecological processes like marine management is always in flux as the diversity of interests and stakeholders shifts through time, mitigating against a single, stable, and widely acknowledged interpretation. Although certain accounts may stabilize and emerge as the official version adopted by policy makers, CBMRM overflows its boundaries, and is instituted by local actors in variable ways and across diverse domains encompassing ecology, marine livelihoods, culture, and politics. Arguably, a detailed account of these local interpretations of CBMRM is paramount for designing adaptive management regimes which can adjust both to biological and social dynamics (Folke et al. 2005; Lemos and Agrawal 2006). However, the main line of argument we wish to develop is that a careful examination of how stakeholders construe the success or failure of CBMRM initiatives in which they engage—or against which they resist—provides an unprecedented opportunity for revealing generalizable patterns of how environmental management policies are received, negotiated, and repurposed by actors.

Below we focus on an empirical case study of Moorea’s PGEM. Implemented in 2004, the PGEM was initially conceived as a decentralized marine spatial planning regime which, through time, increasingly promoted community involvement and stewardship over marine resource governance and management. Moorea’s PGEM went through a lengthy participatory revision process from 2016 to 2021 and concomitantly gave rise to vibrant citizen engagement and overt political contestation that we have documented through ethnographic fieldwork from 2018 to 2021. We document how local authorities, scientists, local environmental and cultural activists as well as fishers brought the PGEM into existence, with some groups touting its success and others its failure. We also analyze the political dimensions of marine governance and how the design and revision of the PGEM emerged through the intricate interplay between local stakeholders, the municipality, and the French Polynesian Government. Finally, we examine how the revision of the PGEM gave rise to shifts, among stakeholders, in the distribution of authority, legitimacy, and expertise in the context of local cultural revival of Polynesian culture and neo-colonial political contestation.

## Study site

Moorea is a high volcanic island located 20 km west of Tahiti, home of the capital of FP, Papeete. As a French Overseas Territory (*Collectivité d’Outre-Mer*), FP depends on the French state for its military defense, foreign affairs, higher education, and monetary policy. From 1984 to 2004, it has gained in autonomy from the French state. FP parliament and government have jurisdiction over the local economy, cultural affairs, as well as the management of terrestrial and nearshore environments.

**Fig. 1:**
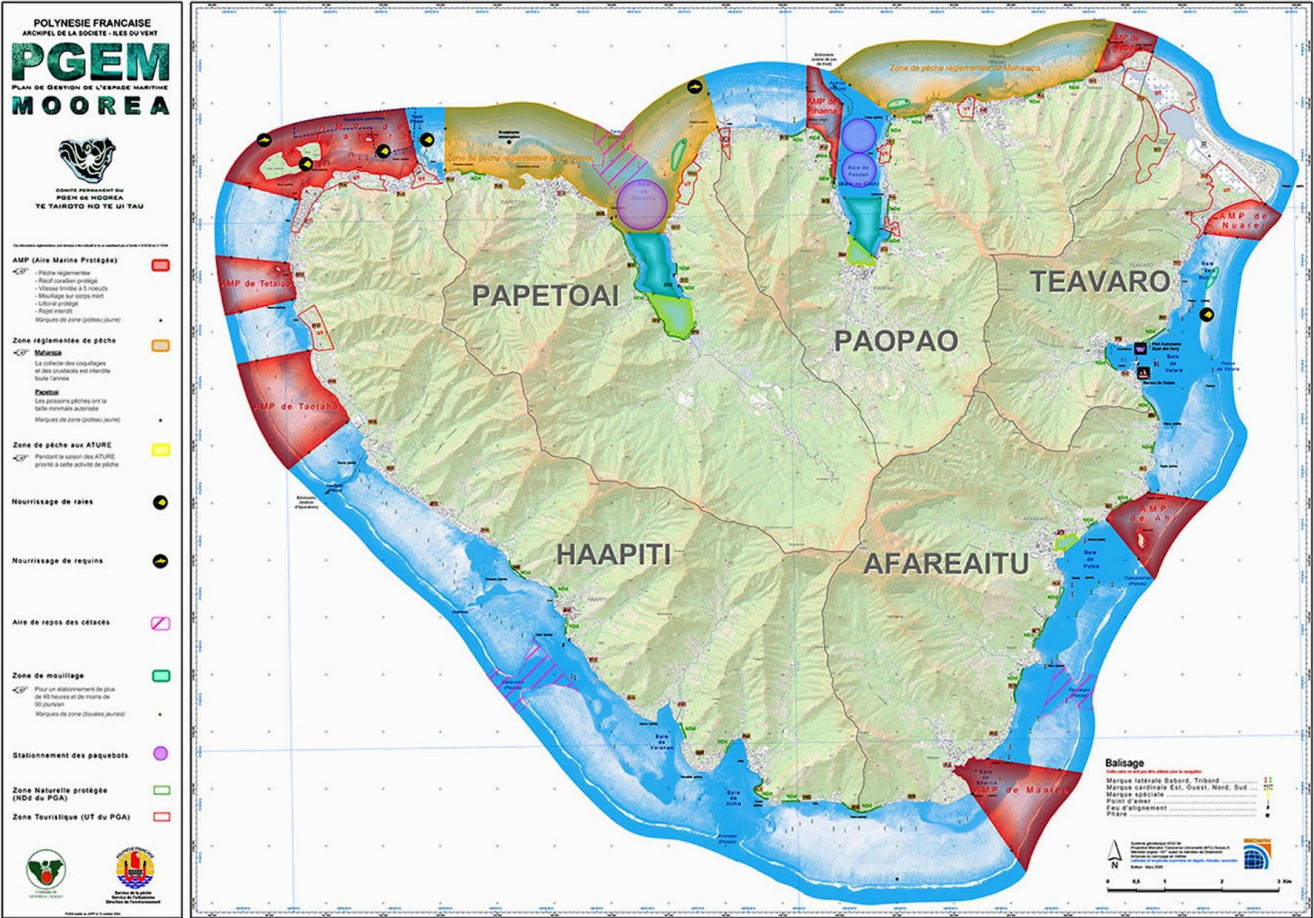
Map of the initial PGEM (2004). MPAs are indicated in red. The orange areas are regulated fishing zones (with species or size restrictions of fish and invertebrates). *Source* Service de l’Aménagement et de l’Urbanisme – Polynésie Française

Moorea along with the small Island of Maiao forms the municipality of Moorea–Maiao. The Island of Moorea is further subdivided into five districts: Afareaitu, Haapiti, Paopao, Papetoai, and Teavaro (Fig. 1). It is the second most populated island of FP (17 463 inhabitants in 2017) and the second most visited in terms of international tourism (IEOM 2017; ISPF 2017). These figures, however, do not account for the importance of local tourism driven by the influx of residents from the more urban Tahiti visiting Moorea over weekends and holidays. Moorea’s demography has exploded over the past few decades—its population doubled from 1988 to 2017—as rapid ferry transport has attracted people working in Tahiti to take up residence in Moorea. This, in conjunction with increased tourism-related activities, has led to fast changing environmental conditions due to increased anthropogenic pressures on both marine and terrestrial environments (Calandra et al. 2021; Loiseau et al. 2021).

Reef and lagoon fishing is an important aspect of local Polynesian lifestyles. Over 50% of households engage in the reef fishery with varying degrees of investment (Rassweiler et al. 2020). Even though only a handful of artisanal fishers generate their full income from reef fishing, it constitutes, for many families, an important buffer for subsistence purposes whether it be through household consumption or the marketing of reef fish (Leenhardt et al. 2016). Reef fishing encompasses a broad diversity of techniques and gears (e.g., line, net, and spearfishing as well as invertebrate harvesting) and a wide array of reef fish species are targeted. Reef fish—often referred to as *i’a tahiti* (litt. Tahitian fish)—are a cultural keystone of Polynesian society as they are an essential component of the local gastronomy and identity. The potential for conflict-use between tourism- and fishery-related activities is essential to understand the socio-political dynamics at play in the management of Moorea’s nearshore marine environment.

Two scientific institutions renowned worldwide in the fields of coral-reef ecology are located on Moorea: the French research station CRIOBE (Centre de Recherches Insulaires et Observation de l’Environnement) founded in 1971 and the American University of California Gump research station established in 1985. Both institutions host marine biology programs producing long-term longitudinal time series about Moorea’s marine environment and biodiversity, and their engagement with local communities ranges from the provision of scientific expertise to local marine managers to the implementation of outreach events destined to youths and residents.

## Methods

To document the PGEM revision process, the resulting socio-political dynamics, and stakeholder perceptions of local marine management, we draw upon ethnographic fieldwork carried out in Moorea from April 2018 to September 2021 focusing on reef-fishing practices, local ecological knowledge, and perceptions of marine management. We attended a total of 53 meetings dealing either specifically with the PGEM revision or more broadly with CBMRM in FP and across the South Pacific (Table 1). Meeting discussions and participant interactions were documented in detail. Moreover, each meeting provided the opportunity to conduct informal discussions and open-ended interviews with the different participants. Finally, through daily engagement with fishers while being embedded within the local communities for over four years, the lead author had the opportunity to gain insight into the socio-political positioning of the most active stakeholders engaged in—or opposing—the PGEM revision. All quotations have been translated by authors from French or Tahitian to English and are reported in Table S1.

**Table 1:** Meetings lead author attended and which were described ethnographically. Attendants were categorized according to the institutions or stakeholder groups they represented

	*N*	Municipal councilmen	PGEM staff	DRM	Other FP agencies	Fishers	Environment. and cultural activists	Scientists	Tourist operators
CLEM Meetings	2	X	X	X	X	X	X	X	X
PGEM Steering Committee Meetings	4	X	X	X	X	X	X	X	X
PGEM Revision Public Meetings	12	X	X			X	X	X	X
PGEM Revision Stakeholder Workshops	5	X	X			X			X
Fishing Committees	12		X	X		X		X	
Meetings organized by local activists and fishers	12					X	X	X	X
Sub-Regional South Pacific Workshop on CBFRM	6			X				X	

## The need to revise

The concept of the PGEM arose in the mid 1990s as a legal decentralization framework developed by the FP government. It was designed to empower local municipalities and communities in the management of their lagoons and coasts which—as part of the public marine domain—normally fall under the sole jurisdiction of the FP government (Cazalet 2008; Calandra et al. 2021). The PGEM was conceived of as a way to meet both France’s commitments to increase the coverage of its marine-protected areas (MPAs) in mainland France and overseas as well as FP’s policy to promote the country’s tourism industry by advertising the beauty of its marine environments (Poirine 2010).

As a holistic marine spatial planning framework, designed by FP’s Department of Urban planning (SAU), the implementation of a PGEM requires the involvement and collaboration of numerous state agencies and local stakeholders, such as the aforementioned Department of Urban Planning (SAU), the Environmental Agency (DIREN), and the Fisheries Department (DRM), alongside local stakeholders and municipal government. While several islands across FP (e.g., Fakarava, Bora Bora) had been identified as target areas for the implementation of a PGEM, Moorea is presently the only island where a PGEM has been fully operational. The design process was initiated as early as 1994, and it took government officials ten years to finalize and enact Moorea’s PGEM in 2004. Among the numerous institutions and stakeholders involved, guidance provided by both the FP Fisheries Department and the CRIOBE Research Station played a pivotal role in the design and completion of Moorea’s PGEM. The initial goals of the PGEM were manifold and included spatially regulating lagoon-based activities (whether recreational or subsistence-based), protecting coral-reef habitats and spawning areas as well as alleviating fishing pressure on the marine resources (Aubanel et al. 2013). The centerpiece of the original PGEM consisted of eight permanent MPAs and two regulated fishing zones (Fig. 1). Regulations covered a wide array of activities including anchoring, navigation speed, seawall construction, land reclamations, recreation, and fishing activities.

The PGEM is governed by a steering committee—including representatives from the civil society, municipal authorities, and central government (Table 2)—which examines any new lagoon-based activities, projects, or developments before their petitioners seek authorizations from the FP government. The effective day-to-day management is operated, on the one hand, by a specifically appointed team of municipal staff members (hereafter, referred as the ‘PGEM staff’) and, on the other, by a local NGO named *Association PGEM* founded by local community members who had proven very active in the past in both cultural and environmental associations.

**Table 2:** Composition of the initial and revised PGEM Steering Committees and of the Comission Locale de l'Espace Maritime (CLEM) which is the appointed committee to steer the revision process

	Initial PGEM Steering Committee		Revised PGEM Steering Committee		CLEM (revision governance organ)	
	Members	No. of votes	Members	No. of votes	Members	No. of votes
Municipality	Mayor of Moorea	1	Mayor of Moorea	1	Mayor of Moorea	1
	5 District Mayors	5	5 District Mayors	5	5 District Mayors	5
Fishery	1 Rep. of reef fishers	1	5 Rep. of reef fishers	5	5 Rep. of reef fishers	5
Tourism industry	1 Rep. of the hotel industry	1	5 Rep. of the tourism industry	5	3 Rep. of the tourism industry	5
	1 Rep. of recreational activities	1			1 Rep. luxury hotels	
					1 Rep. small and family owned hotellery	
Civil society	–	–	–	–	1 Rep. of the Association PGEM	1
Culture	1 Rep. of cultural organizations	1	1 Rep. of cultural organizations	1	1 Rep. of cultural organizations	1
Environment	1 Rep. of environmental organizations	1	1 Rep. of environmental organizations	1	1 Rep. of environmental organizations	1
Science	1 Rep. of scientific institutions	1	1 Rep. of scientific institutions	1	1 Rep. of scientific institutions	1
French State	–	–	–	–	1 Representative of the French State	1
FP government	–	–	–	–	1 Rep. of FP Parliament	1
FP agencies	1 Rep. of Fisheries Service (DRM)	1	1 Rep. of Fisheries Service (DRM)	4	1 Rep. of Fisheries Service (DRM)	4
	1 Rep. of Urban Planning (SAU)		1 Rep. of Urban Planning (SAU)		1 Rep. of Urban Planning (SAU)	
	1 Rep. of Environment Agency (DIREN)		1 Rep. of Environment Agency (DIREN)		1 Rep. of Environment Agency (DIREN)	
			1 Rep. of Maritime Affairs (DPAM)		1 Rep. of Maritime Affairs (DPAM)	
Other FP agencies	–	–	–	–	7 Rep. of other FP Agencies^†^	7
Total	15	13	23	23	27	27

^†^Seven represented agencies: Chamber of Commerce and Industry (CCISM); Chamber of Agriculture and Reef Fishery (CAPL); Land Ownership Agency (DAF); Public Equipment (Direction de l'Equipement); Youth and Sports Agency; Tourism Agency; Rural Development Agency

After ten years of existence, the municipality and FP government decided to revise the PGEM. They initiated in 2015 a wide-scale participatory campaign which gave birth to a revised version of the PGEM enacted by the FP government in September 2021. The revision process was governed by an appointed committee (CLEM—Commission Locale de l’Espace Maritime—Table 2) and executed in the field by the PGEM staff.

The recognition that the PGEM needed to be revised begs the question of how the original PGEM came to be perceived as unsuccessful. The initial diagnosis for the need to revise the PGEM came from within the steering committee as early as 2010. Committee members were concerned by the growing number of lagoon-based activities, the lack of sufficient means to enforce existing regulations and the need to secure greater engagement from stakeholders. In 2013, as the PGEM was approaching its 10th anniversary, a local group of stakeholders—named MAMA—involving PGEM staff, scientists, environmental activists, and representatives from the FP agencies started meeting on a regular basis to sketch out guidelines to prepare a full-blown revision. Desiring to carry out the revision through a wide-scale consultative and participatory process, the FP government and MAMA group sought the assistance from a European Union-funded project—operated by the Pacific Community (SPC^1^)—focused on the management of natural resources and the resilience of coastal environments across the Pacific (RESCCUE^2^). This created the opportunity to access both funding and expertise from a new set of actors—namely social scientists specialized in participatory framework designs—to aid in the diagnosis of the failed project and to guide the revision process towards a new version of success. The official diagnosis of failure as detailed in a RESCCUE report was that the PGEM lacked “legal, financial, and human assets” and had been severely criticized by local stakeholders, most notably fishers who argued that they were not fully consulted about the implementation of the MPA network (Narcy and Herrenschmidt 2014).

In our interviews, PGEM staff indicated that they were convinced that the original PGEM had failed to achieve its goals. They voiced concerns about the increasing number of activities in the lagoon over the ten years since the PGEM’s implementation and reckoned that the initial PGEM scheme lacked the capacity to manage emerging diverse interests (Quote-1—Table S1). Similar to the RESCCUE report, PGEM staff also pinned the project’s failure on fishers’ lack of engagement and compliance as well as their overt opposition to the management scheme (Quote-2—Table S1).

The lack of positive ecological results was also a crucial element used to compose the official version of failure. For instance, government officials told us that the PGEM “is not working, fishers are not complying, and ecological results are minimal.” Indeed, an ecological study (Thiault et al. 2019) had shown that the biomass of harvested fish species was slightly higher on average inside MPAs where fishing was entirely prohibited but that this increase was weak compared to those documented in other published MPA analyses. According to the authors, it was the absence of fisher compliance, the lack of surveillance, and the “limited public appreciation about the benefits of MPAs” that were key reasons for the “limited ecological benefits” of the PGEM (Thiault et al. 2019, p. 8). The actions of the discontent fishing communities and scientists’ findings were now aligned, moving in the same direction, yet for different reasons, and pointing to failures of the PGEM.

## Fishers’ contestation of the PGEM

Despite the official praise of the PGEM during its early phases (Conseil des Ministres 2021, p. 22269), Moorea’s fishers had never been fully enrolled in the project idea. In fact, some fishers actively protested against the project as it was being officially established (Gaspar and Bambridge 2008; Walker and Robinson 2009). The fishing communities’ widespread criticism inhibited the original PGEM from ever becoming fully stabilized as a “success” across all the different interests.

While most of the fishers with whom we have interacted since 2018 agree that some form of marine management should be undertaken, they argued that the PGEM was flawed and unjust. The most common discourse was that the PGEM was geared towards the promotion of tourism at the expense of fishers. In meetings, fishers regularly indicated that the MPAs were opportunistically located near resorts, or they disgruntledly complained that regulations were only enforced for fishers while non-complying tourist operators were never sanctioned (Quote-3—Table S1). Fishers also frequently mentioned how permanent no-take zones were intrinsically unfair, forcing fishers living onshore from MPAs to travel greater distances to reach legal fishing grounds.

Several social-science studies articulated these concerns, providing further legitimacy to fishers’ interpretations of the PGEM. Walker and Robinson (2009) detailed a concern described by a renowned local cultural activist (Quote-4—Table S1) about how the PGEM had displaced essential subsistence and cultural fishing activities carried out by women. In addition, Gaspar and Bambridge (2008) wrote how the PGEM had been constructed around technocratic and scientific principles which displaced Polynesian territorial understandings. Indeed, the spatial zoning of the lagoon alone—disconnected from terrestrial issues—perpetuated the land/sea divide the FP administration inherited from French colonial rule which contrasts with Polynesian forms of management embracing both land and sea in continuous territorial units. Moreover, the French name of the PGEM as well as its technocratic jargon has pushed Moorea stakeholders to conceive of it as an extension of French rule (Quote-5—Table S1) and, hence, as a governance mechanism imposed from the outside displacing Polynesian identities and modes of being (Quote-6—Table S1) (Rigo 2004).

Although efforts were made to consult with fishers during the initial design of the PGEM, those who acknowledged having been consulted argued they had been duped and stated they were promised non-permanent closures (*rāhui*—see below) instead of MPAs (Quote-7—Table S1), even though the PGEM staff rejects that such promises were ever made. Arguably, this situation reflects more than a misrepresentation of MPAs or a problem of translating from French to Tahitian, but rather an attempt by Moorea’s fishing communities to institute a different version of marine management, one over which outside experts would have less control.

## Total failure?

In contrast to many fishers, environmental and cultural activists on Moorea framed the initial PGEM as a success in checking the overdevelopment of the tourism industry in Moorea. They have argued that it addressed their demands of gaining greater decision-making power in overseeing developments on the island’s public marine domain. The PGEM Steering Committee’s environmental NGOs representative argued that the PGEM provided an unprecedented arena in which the voice of citizens, through their representatives, could be heard by the FP government (Quote-8—Table S1). The number of projects declined (over 200 demands ranging from seawall construction to new nautical recreational activities submitted for approval by residents or local businesses) by the PGEM Steering Committee over its first ten years of existence is used, by PGEM advocates, as a metric of success. The role of the PGEM as a counterweight to the FP government’s desire to support growing tourism development on Moorea is solidly anchored into the foundational struggle—which occurred in 2000—of residents against the proliferation of over-water resort bungalows. Those who had led this struggle are now the PGEM’s fiercest advocates. One of them repeatedly declaimed a narrative of this struggle during several PGEM revision meetings to remind attendants they had the power to oppose projects coming from the top (Quote-9—Table S1). The recursiveness of the narrative compels us to consider it as having become, for many local activists, part of the founding mythology anchoring the PGEM as a way to empower local citizens *vis-à-vis* the FP government.

However, the environmental and cultural activists tempered their interpretations of PGEM success by misgivings about the steering committee’s decision-making authority arguing that the committee should have more than a simple consultative voice. They would wish for the committee’s decisions to be final to avoid the ability of FP agencies to overrule them. But this sense of failure has often been nuanced as many committee members pointed to the fact that FP agencies had rarely overruled their decisions (Quote-10—Table S1). However, the interpretation of the PGEM as a counterweight to policies implemented by the FP government depends on an alignment between the interests of local civil society organizations and of the municipality. In the absence of such an alignment—as illustrated by the latest developments around the final enactment of the revised PGEM (Appendix S1)—these interpretations of success may shift, jeopardizing the very existence of the PGEM.

Activists suggested that the initial PGEM escaped the control of the FP government by presenting obstacles to central government-imposed policies and by coercing inter-agency collaboration. One of the PGEM’s supporters who had actively participated in the revision process from the beginning claimed that some FP agencies had hoped, and even planned, for the PGEM revision to fail in order to revoke the PGEM entirely in favor of other single-agency piloted legal frameworks geared towards more-specific environmental or fisheries-related purposes. He also argued that the FP government actively sought to undermine the revision process by lending a friendly ear to the *Association Rāhui* whose members were the fiercest detractors of the PGEM.

## Rāhui: All in favor?

The dissatisfaction with permanent MPAs and the demand for rotational closures—inspired by the principles of *rāhui*—have been the most prominent alternative vision of marine management on Moorea. *Rāhui* refers concomitantly to territorial units—pie-shaped territories running from mountain ridge to reef crest—and a form of natural-resource management placing specific species or spaces under a temporary harvest ban (Bambridge 2016).

In pre-contact Tahiti, estates were governed through a nested hierarchy of nobiliary elites who had the power to establish a *rāhui* on the territory or resources they controlled. The notion went hand in hand with that of *tapu*—a strong spiritually sanctioned prohibition—under which resources could be placed for the duration of the *rāhui*. Most often, *rāhui* were destined to replenish marine or terrestrial resources in view of their future use for specific religious and political ceremonies. The institution was progressively undermined by Christianization and colonialization (Bambridge et al. 2019).

Parallel to the Pacific-wide ‘renaissance’ of customary forms of marine management (Johannes 2002), the concept of *rāhui* has reemerged across FP in the past decades under the double influences of Polynesian cultural revival movements and the state-led promotion of CBMRM initiatives (Fabre et al. 2021; Filous et al. 2021). The most visible case of *rāhui* has occurred in Teahupoo where it was implemented in 2014 and which has been framed—by community members, local media, and government authorities—as a success both in terms of stakeholder engagement and ecological outcomes^3^ (Fabre 2021).

The growing demand for the implementation of *rāhui-*inspired forms of management in Moorea has been expressed most clearly by the work of the *Association Rāhui*, founded by residents from the district of Haapiti in 2016, who vociferously contested the PGEM and promoted *rāhui* as an alternative (Hunter et al. 2018; Fabre 2021). Their main lines of argument captured the key grievances voiced around the island that the PGEM was an institution geared towards the promotion of tourism at the expense of fishers, residents, and the marine environment (Quote-11—Table S1) while “lacking transparency and shared governance.”

The *Association Rāhui* proposed eliminating the PGEM and in its place establishing in each of Moorea’s districts, *rāhui* committees named *toohitu* (litt. council of the seven) composed of fishers and community leaders. In other words, they were inventing and defining new groups and endowing them with new goals in their attempts to fail the PGEM. These committees would oversee the management of their district’s lagoon and implement rotational, rather than permanent, closures according to their expertise. The idea to implement *toohitu* committees was a way to root their project in Polynesian tradition while promoting what the *Association Rāhui* understood as a more democratic mode of governance. The Association’s shrewd deployment of the concept of *rāhui* to enroll a diverse coalition of interest groups provides an insightful example of the dynamism and plasticity of ‘traditional’ concepts. On the one hand, while *rāhui* was, in the past, nested in the strict hierarchies of the pre-contact socio-political order it becomes, in the present, a flagship of democratic governance. On the other hand, the institution of the *toohitu* originated as a post-contact form of governance promoted by Christian missionaries as a way to downplay the political power of Polynesian nobiliary elite (Saura 1996).

For the *Association Rāhui*, the notion of *rāhui* was used as a means of political contestation against the municipality by seeking to transfer decision-making power to local fishers and residents. The ‘Polynesianess’ of the *rāhui* concept was also employed in contrast to two of the most negatively evaluated effects of the colonial and post-colonial order: money and profit, each portrayed as the essential motives behind the initial PGEM (Quote-11—Table S1). Indeed, equating indigenous identity with non-capitalist modes of being is a well-documented and effective strategy deployed by indigenous people around the world when they seek to assert their political will (Kuper 2003; Dove 2006).

The Association’s concerns gained relevance during the revision process as they managed to align the interests of fishers, church pastors, and many community leaders and tie them together through an appeal to Polynesian identity. Moreover, the Association’s members were adept political operators and, seeking to circumvent the revision process, lobbied FP government officials to endorse their project. However, as the PGEM revision proceeded, the Association progressively lost steam and suspended their activities, in 2020, as its core members invested their efforts (unsuccessfully) into direct political action by running as candidates during the municipal elections. Nonetheless, the Association’s activism engaged many different interest groups, including the PGEM staff in charge of the revision, in the idea that implementing district-level fishing committees was a pathway to secure greater engagement of fishers into the PGEM. Even though the Association’s life was short lived, they were able to influence the PGEM revision towards a version of success that drew on the revitalization of Polynesian culture and identity and a long-festering sense of neo-colonial dispossession and acculturation.

## Cultural revival and neo-colonial contestation

The appeal of *rāhui* in Moorea was not only tied to its invocation of a governance design that devolved greater power to residents and fishers, but also to the concept’s links to Polynesian culture, identity, and cosmogonies. Two related concepts—*tapu* and *mana*—were frequently deployed by many community members as a means to provide *rāhui* with greater legitimacy than a “disenchanted” PGEM. For some, *rāhui* would be “self-enforced” due to the spiritual sanctions that would befall those breaking the sacredness of *tapu* instituted by a *rāhui* (Quote-12—Table S1).

The spiritual dimensions of *rāhui* are further revealed by its connections with the notion of *mana* found across the Austronesian world which is fundamental to political and religious authority, and that can be defined as an expression of power channeled by skilled practitioners vis-à-vis spiritual or godly entities (Keesing 1984) (Quote-13—Table S1). These core Polynesian concepts are not only widely accepted among fishers and residents on Moorea, but they also have traction among many municipal and FP government officials who identify as Polynesians.

Even though most officials expressed their cultural attachment to these Polynesian concepts, they also stated that they no longer apply in the contemporary context and were not incorporated into the original PGEM design (Quote-14/15—Table S1). That the original PGEM was not developed around *rāhui* appears to have been a strategic mistake and this resulted in failure to gain sustained and widespread support for the management initiative. In contrast, in Teahupoo on the south-eastern tip of the island of Tahiti, community members and FP agencies from the onset explicitly conceived of the marine management as *rāhui* and the management scheme has widely been acknowledged as a success both for the FP government and among stakeholders (Fabre et al. 2021).

**Fig. 2:**
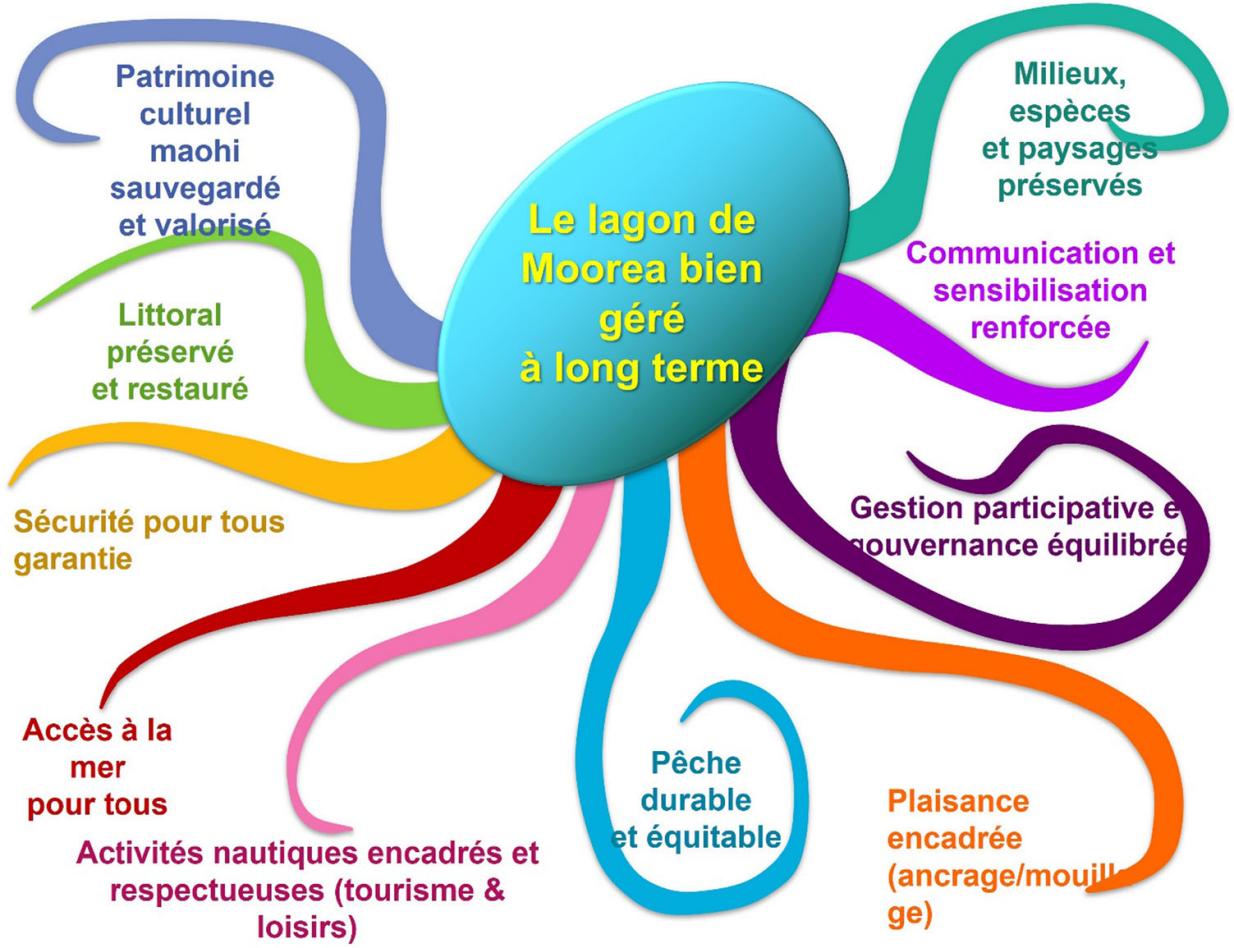
Goals defined by PGEM staff through consultative and participatory workshops. The ten identified goals, represented as a ten-tentacle octopus, cover a range of objectives: (i) regulation of specific activities (sustainable and equitable fishing, mindful recreational nautical activities, regulating sailboat), (ii) reaching island wide socio-ecological goals (promotion of local culture, conservation of the coast, marine species and marine landscapes, users’ safety, and access to sea) and (iii) implementing collaborative governance (participatory management and reinforced communication). *Source* RESCCUE Project

Despite the PGEM’s evident techno-scientific imprints, the architects of the original PGEM tried to weave in cultural meaning and representations by tasking the steering committee’s representatives of cultural and environmental associations with the design of the PGEM’s logo and motto. An octopus, a widely known mystical being in Polynesian cosmology, was chosen as the logo (Figs. 1, 2). The sea creature’s tentacles are understood as the eight main valleys of Moorea, and more broadly, it is known to be hewn from an amalgamation of marine beings, humans, and *Ta’aroa*—the god considered as the creator of the world and of all the spiritual and living entities (Gaspar and Bambridge 2008).

Rather than just an apolitical spirit being, Moorea’s mythical octopus also encapsulates the life-destroying consequences of colonialism. It describes how the arrival of foreigners had disrupted the social harmony around the island and caused the angered octopus to severe its relations with the communities who had abandoned their lifestyles when welcoming the newcomers (see Gaspar and Bambridge 2008 for a detailed account of the legend). The octopus being was invoked during the implementation of the initial PGEM to restore harmony and balance to the island. More than mere tokenism, it was called upon to facilitate and compose the success of the PGEM by renowned cultural activists who had been active, before the implementation of the PGEM, in several struggles against tourism-oriented development projects in Moorea and who had become some of the original PGEM’s strongest supporters.

## Stakeholder consultation

A central-guiding principle of the PGEM staff’s initiative to revise the PGEM was stakeholder consultation, which they envisioned as a process where they could redefine the goals and priorities of the revised PGEM that would better align with community interests (Quote-16—Table S1). For this reason, PGEM staff, aided by “participatory conservation experts” hired through the EU-funded RESCCUE project, systematically kicked off their meetings with a PowerPoint image representing the different activities they had identified and that the PGEM sought to regulate: over-water resort bungalows, scuba diving, jet skiing, ray feeding, snorkeling, and fishing. With these uses as the central focus, a synthesis of the PGEM’s objectives emerged from consultation workshops held from 2016 to 2017 and was codified as a central framework of the revision (Fig. 2). Again, adopting the octopus and its spiritual connotations, the entity was a key stabilizing device deployed by the PGEM staff to align and assemble the heterogenous and cross-cutting interests and assert their interpretation of how the PGEM would be revised.

**Fig. 3:**
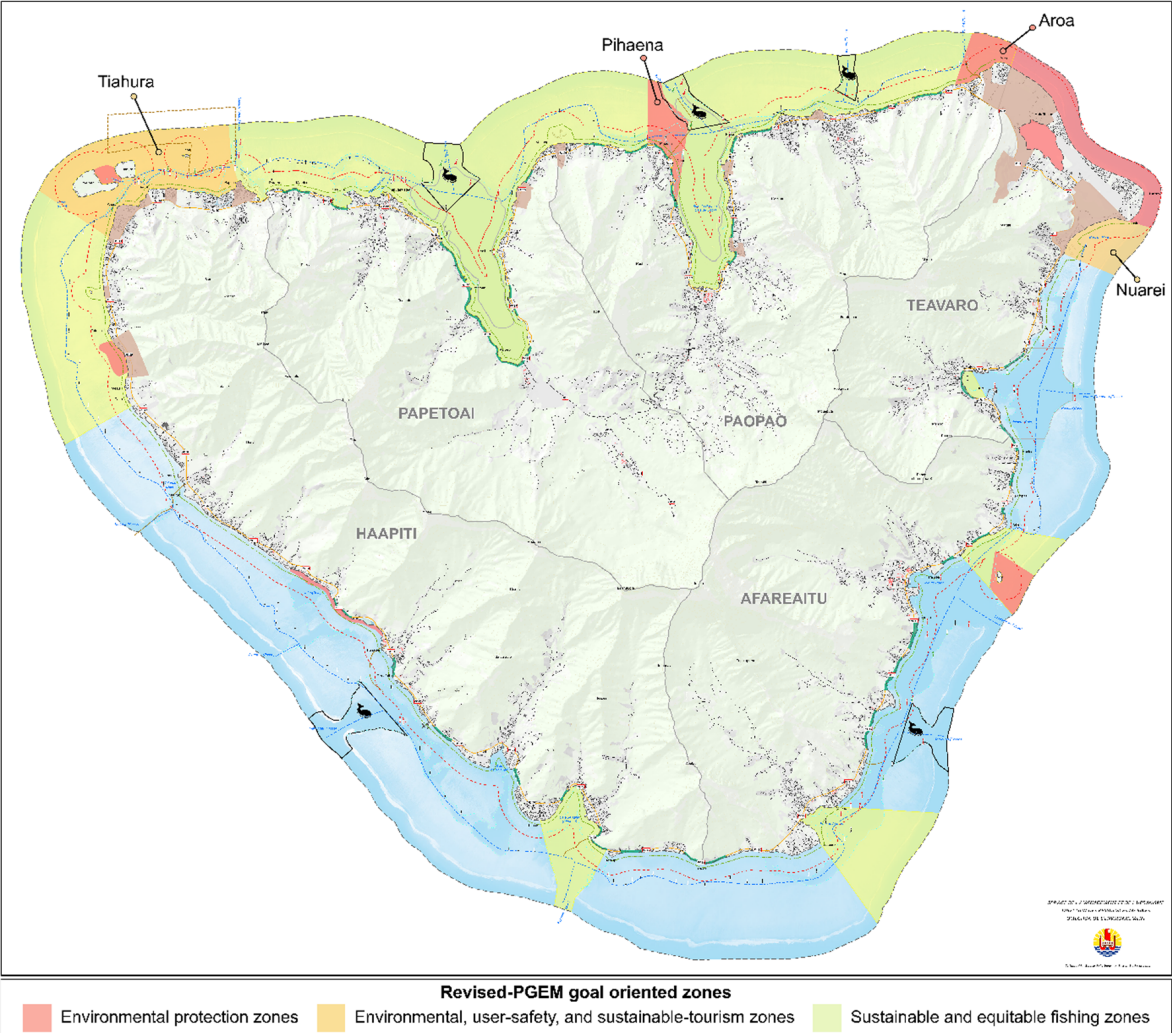
Goal-driven zones of the revised version of the PGEM. The spatial representation of the PGEM shifted from a unified map (Fig. 1) in which the MPA network is the main feature to a set of four maps: one representing the main goal-oriented zones (below), one representing the specific zoning for fishing, another representing the recreational activities’ zones and a last one combining the previous three. *Source* Service de l’ Aménagement et de l’Urbanisme – Polynésie Française (legend and zone names have been added by authors)

To address the 10 identified objectives, the PGEM staff proposed to create three kinds of goal-oriented zones (Fig. 3): ‘Environmental protection zones’; ‘Environmental, user-safety, and sustainable-tourism zones’; ‘Sustainable and equitable fishing zones.’ The staff instituted an important shift in vernacular by dropping the term “MPA,” which in the original PGEM signified no-take areas. Four of the initial PGEM’s MPAs were instead relabeled as two ‘Environmental protection zones’ (Fig. 3—Aroa and Pihaena zones) and into two ‘Environmental, user-safety, and sustainable-tourism zones’ (Fig. 3—Nuarei and Tiahura zones). Both of the new zones effectively ban fishing while not portraying the ban as the main goal. The remainder of the previous MPAs have been transformed or reshaped into Marine Managed Areas (MMAs) labeled as ‘Sustainable and equitable fishing zones’ in which regulations would be defined by fishing committees and the DRM (FP Fisheries Department) working in parallel to the PGEM Steering Committee.

Ostensibly defining the goals through stakeholder consultation and shifting the core vernacular reflect the PGEM’s choice to reconfigure the balance of power among stakeholders and the degree to which the various interests weighed in the design of the new PGEM. Transforming MPAs into MMAs was a critical conceptual shift as it put greater emphasis on the roles of fishers and community members in the governance regime while displacing the position of natural scientists, who had been the strongest advocates for strict MPAs and who equated “success” with biodiversity protection.

## Inventing institutions

Aware of the fishers’ widespread criticism of the initial PGEM, the PGEM staff placed a high priority on fisher participation. To do so they created fishing committees in each district and opened them to all fishers—regardless of their investment in the fishery—to design, in consultation with DRM, fishing regulations to be applied in each district’s lagoon. The design of the district-level committees emerged progressively during the revision as a direct outcome of the involvement of burgeoning local fisher groups, including the *Association Rāhui*,^4^ which surfaced from 2016 to 2017 in three of the island’s districts (Teavaro, Paopao and Haapiti). The invention of these new institutions was an attempt to align the heterogenous interests of the fishing communities. Of course, creating new institutions by writing them down as words in the revised project documents is easier than forming them on the ground, let alone controlling them.

**Fig. 4:**
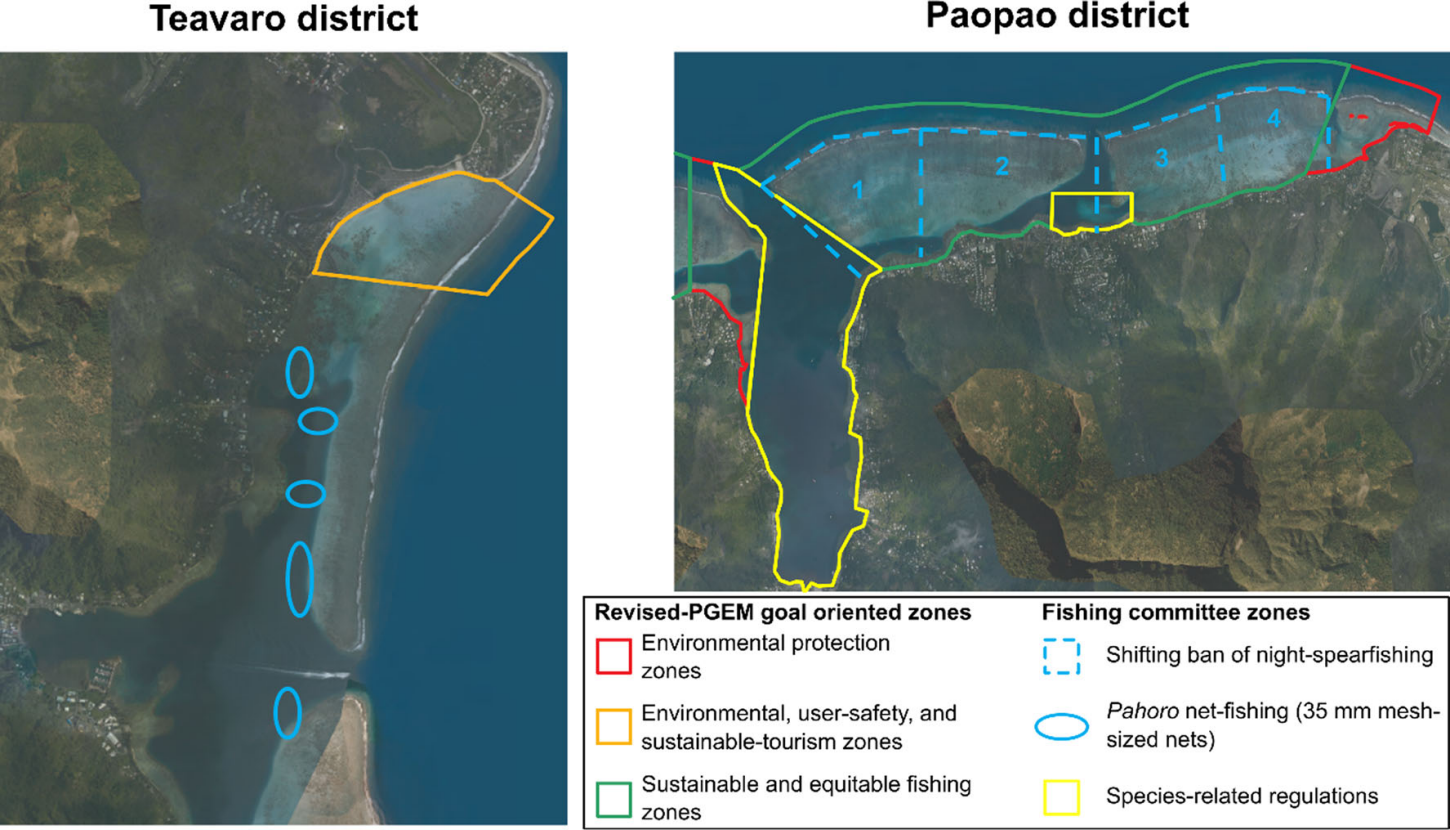
District-level fishing committee decisions for the districts of Paopao and Teavaro. *Source* DRM

The first fishing-committee meetings were convened by DRM in late 2017. The results were mixed and the degree of investment of fishers varied greatly around the island. In Haapiti and Papetoai where PGEM contestation was strongest, fishers’ participation did not take root before the final months of the revision in 2021. In Paopao and in Teavaro, however, the presence of recently formed fisher groups led to innovative fishing regulations. Rather than passively enrolling themselves in the new fishing committees, Paopao and Teavaro, borrowed the idea and directed the committees towards their own goals. In 2017, the Teavaro committee asked the DRM to organize a meeting with fisheries scientists to discuss minimal sizes of harvested fish. The workshop attracted dozens of fishers who brought along fish they had caught earlier the same day so that they could be measured. One of the fishers’ goals was to demonstrate that a cultural keystone species, *pahoro*—initial phase parrotfish—caught with smaller mesh-sized nets than the 40 mm FP-wide legal minimum had reached sexual maturity warranting that they could be harvested without endangering the species’ healthy reproduction. The workshop’s outcome was, for participating fishers, a complete success as they secured from DRM the promise to make an exception for Moorea allowing the use of 35 mm mesh-sized nets for *pahoro* fishing. DRM requested that fishers define a select number of spatially delineated areas where this type of fishing could take place (Fig. 4) in order to ensure tight surveillance of the use of these nets. Through these workshops the Teavaro fishers aligned fish and scientists with their own interests to keep harvesting *pahoro*.

The Paopao fishing committee also developed a new inner-lagoon management plan. Its participants were fierce detractors of night spearfishing, considering it as the most deleterious fishing practice, while acknowledging its importance for many unemployed families and youths. Instead of trying to ban the practice altogether, the committee designed a system of rotational closures. Two pass-to-pass lagoons were divided into four distinct areas among which one area at a time would be closed to night spearfishing (Fig. 4). The ban would then shift, every 2 years, from one zone to the other, moving eastward, forming a full cycle over a period of 8 years. The outcome of the Paopao fishing-committee meetings emerged as a lynchpin for DRM staff to construe the revision process as a success because they were progressively managing to enroll fishers in the revised version of the PGEM and to gain their trust (Quote-17—Table S1).

The outcomes achieved by the Teavaro and Paopao committees were leveraged by the PGEM staff to invigorate fishing committees in other parts of the island. For example, during the January 2019 Afareaitu fishing-committee meeting participation was dismal and only five fishers from the district were present. A DRM agent led the meeting and participating fishers nodded their way throughout the PowerPoint presentation of the fishing committee governance regime to be enacted. No negotiations or debate had taken place and the meeting’s outcome offered nearly no change to pre-existing regulations.

Two years later, in February 2021, however, we attended the last fishing-committee meeting held before the revised-PGEM’s finalization and the outcomes were radically different. Over 20 fishers participated. After the DRM agent’s presentation of the district-level regulations to be implemented in the revised PGEM, heated debates took place as fishers indicated that nothing had changed compared to the initial PGEM regulations. But after presenting the designs from the Teavaro and Paopao fishing committees, a real process of negotiation started between participants and DRM agents.

The most interesting debate surrounded demands from fishers to open the Afareaitu MMA (previously an MPA) to day-time spearfishing. Despite the DRM agent insistently advising against such a decision, the last word was given to fishers and the decision to allow day-time spearfishing was finally enacted to the great surprise of most participants (Quote-18—Table S1). This illustrates how the authority granted to fishing committees presented a clear break from the early PGEM governance, but also how bureaucratic control over the committees was limited (Quote-19—Table S1). An institution created by the PGEM staff was overflowing its envisioned boundaries and being repurposed in ways that was forcing the PGEM staff to cater to the interests of the fishing committees.

Despite the growing engagement of fishers in the fishing committees, overall participation of—and representation of—fishers was problematic for the PGEM staff. “Representation” was a guiding principle of the revision process, but it presented numerous challenges when put into practice (Quote-20—Table S1). Initially the fishing committees’ representatives were handpicked by the municipality, but once the fishing committees started to meet, the appointment of representatives was voted upon at the end of each meeting.

Yet, in some cases, fishers still felt they were not well represented. The case of the Paopao fishing committee mentioned above provides an illustrative example. The committee, piloted mainly by a handful of net fishers, set out to implement strong regulations against night spearfishing. However, night-spearfishers were not engaged in the process and did not attend any of the Paopao district meetings we attended. After one of the district committee’s meetings, we met a young spearfisher from Paopao—whom we had informed of the date and location of the meeting—and asked him why he had not come to the meeting. He replied that he felt his presence was unwelcome due to the scorn committee members demonstrated against night-spearfishers (Quote-21—Table S1). This night fishers’ comments illustrate how a project inevitably has detractors that will assemble potentially destabilizing elements that might eventually fail a project. The solidity of a project is never given in advance but rather an achievement of those who produce its success. The introduction of fishing committees, nonetheless, enabled a shift of power towards fishers. The rebalancing of decision-making power and securing stakeholder engagement have been core processes for crafting the new version of “success” for the revised PGEM.

## Shifting the position of natural scientists

Many in the fishing community were uneasy about the role scientists played in the original establishment of the PGEM. During the revision process there was growing appreciation among fishers and activists for Polynesian ecological knowledge about the lagoon and the possibilities it provided for challenging scientific knowledge (Quote-22—Table S1). For instance, to impose their vision of the PGEM outcomes, the *Association Rāhui* carried out its own “citizen science” underwater fish counts and produced a written report summarizing the ecological state of the lagoon. Although their goal was to challenge the authority of Western science (and its local practitioners), the use they made of some of its methods—underwater fish counts—indicates how they fully recognized the importance of knowledge that is deemed scientific and how policy makers invoke it as a neutral arbiter to assert political authority and legitimacy.

During the revision process, PGEM staff began to recognize the growing local skepticism towards scientists and the knowledge they produce which was grounded in two key concerns. First of which was the near-absence of Polynesian scientists working in Moorea. Second, there was a lack of communication from both the French and American scientific institutions about the many experiments carried out in Moorea’s lagoon. These concerns participated in questioning the authority of scientific knowledge in the new PGEM designs. Furthermore, rather than unbiased objective knowledge about the marine environment that could guide PGEM decision making, scientific knowledge was now being framed as contributing to PGEM’s failure by imposing post-colonial interests at the expense of Polynesian ones.

To neutralize these concerns, PGEM staff recast their relationship with and the position of scientists during the PGEM revision, a shift which was reflected in several aspects. First, natural scientists were progressively sidetracked all along the revision process to the point where they were no longer invited to play a leading, authoritative role in revision workshops and meetings (Quote-23—Table S1). Second, the revised text of the PGEM proposed a regulatory framework for scientific activities: scientists would be required to request approval of their lagoon-based scientific projects from both the PGEM Steering Committee and the district fishing committees. This shift in oversight over scientific activities has been a source of concern for both the CRIOBE and the Gump Research Station fearing that it may constitute a significant impediment to their work. Scrutiny and red tape were not welcomed by scientists. Concern was fueled by the experience of a scientific team which presented one of their proposed projects to Haapiti’s fishing committee but was turned down. The new positioning of scientists vis-à-vis the PGEM was confusing to scientists and contrary to their self-image as neutral, apolitical observers providing impartial data that was immune to scrutiny by non-experts. Now they were cast as just another stakeholder who might be objects of suspicion or trust, strongly unwelcomed or embraced, cast aside or invited to participate (Quote-24—Table S1).

The transformed role of scientists in the revised PGEM was also evident in fishing-committee governance. Even though a representative of Moorea’s two scientific institutions was invited to sit in each of the fishing district committees, neither of the scientific institutions made explicit recommendations nor did the representative participate in the debates during the meetings. Nevertheless, many scientists did have concerns. Some natural scientists thought the new regulations were too complex and consequently would be ineffective. They approached the PGEM staff after the last fishing-committee meeting in early 2021 and asked them for a special, closed-door meeting where they could express their concerns and provide their expertise. The PGEM staff agreed to hold the meeting, but the meeting was not fruitful for the scientists. The PGEM staff regretted that such concerns were not deliberated publicly during the fishing-committee meetings which would have provided fishers with the opportunity to consider, debate, or ignore the position of the scientific community. The attempt of some marine scientists to assert their authority in the mold of the original PGEM by positioning themselves as external actors who provide expertise to local stakeholders was met with PGEM staff’s strategy to solidify their version of the PGEM’s success by blurring the line between scientists and local stakeholders.

## Discussion and conclusion

The revision of the PGEM set the stage for local stakeholders as well as FP government and municipal officials to voice their interpretations regarding the successes and failures of Moorea’s marine governance and management. Drawing from a diverse mix of claims and practical strategies—ranging from the desire to revitalize Polynesian culture and way of life to the necessity to empower local authorities and stakeholders vis-à-vis central government authorities—stakeholders’ interpretations of the PGEM goals varied considerably both across groups and through time and had diverse effects on the revision process. Instead of a smooth object where the strengths and weaknesses of CBMRM are uncontestable and detectable from any vantage point, our account illuminates how the PGEM was an entangled web of dynamic relations where different stakeholders fought to stabilize their interpretations by enrolling various allies and aligning their different interests. The successes and difficulties PGEM staff had when attempting to stabilize the project during the revision process into a cohesive and widely embraced governance scheme highlight the instability of the core concept of co-management or CBMRM initiatives: the concept of “community” which often goes unquestioned. By placing “community” at the center of the decision-making nexus, the PGEM staff confronted the practical difficulties of assuming that local stakeholders form well-defined, homogeneous units “that speak[s] with a single voice” (Watts 1999, p. 37) and that project managers have the capacity to rationally seek solutions that appease all interests. As illustrated in our case study, “communities” are partially composed through the project itself and are in constant motion with cross-cutting social and political intricacies that emerge, shift, and dissipate through time during a project’s life. For these reasons, the questions of representation and legitimacy of community interests appeared as a touchstone of local contestation against the initial PGEM and, consequently, as essential issues to address during the revision. Yet even though such questions have been noted in the conservation science literature (Agrawal and Gibson 1999; Berkes 2010), their deeper political implications and how they may participate in reproducing and generating asymmetries in power distribution or hierarchies in knowledge production systems are often sidetracked in the design and evaluation of marine governance and management regimes (Dressler et al. 2010; Mazé et al. 2017).

As our description of the PGEM revision has illustrated, stakeholders involved in CBMRM invariably mix in their political interests, a point highlighted by Lemos and Agrawal who define environmental governance as: “the set of regulatory processes, mechanisms and organizations through which political actors influence environmental actions and outcomes” (2006, p. 298). Indeed, the devolvement of governance and management involves political positioning as attempts are made to transfer decision-making power from one institution or set of actors to another. The fragility of the tripartite PGEM governance (i.e., civil society, municipal authorities, central government) is a testimony to the importance of considering the political dimensions of marine, and more broadly natural-resource governance. The latest developments surrounding the enactment of the revised PGEM provide a clear case in point (Appendix S1). Yet the success of many CBMRM governance schemes is often constructed around policy makers’ interpretation that political contestation is avoided and that rational, apolitical strategies guided by scientific knowledge will lead to positive outcomes.

However, construing CBMRM as an apolitical, technical activity outside of democratic contestation implicitly positions local stakeholders as subordinates to experts from government agencies, scientific institutions, or conservation organizations (Beck 1992; Mitchell 2002; Eyal 2019). Indeed, the technocratic and scientific imprint of the initial PGEM was met with stakeholders’ overt hostility towards scientific authorities which was fueled by the growing revival of local Polynesian knowledge as a valid domain. The attempts of marine biologists to position themselves as outside observers providing their expertise to stakeholders and policy makers illustrate how conservation initiatives often seek to police a boundary between experts and non-experts (Jasanoff 2004). For without this boundary, the experts’ neutrality becomes a target of skepticism, leading to a questioning of their credibility and legitimacy in the eyes of local stakeholders. Yet as our case study of the PGEM has illustrated, the controversy overflowed the boundary between experts and non-experts, as different stakeholders attempted not only to assert their own authority and legitimate non-expert knowledge as an acceptable guide to policy but also by redefining “marine resource management” as something well beyond just “marine resources” to include politics, identity, Polynesian cosmology, and livelihoods. Even though the PGEM and DRM staff sought to atone this expert/non-expert divide—by sidetracking scientists or by empowering fishers in decision making—they worked to uphold such an epistemological positioning when framing the participation of fishers as ultimately a means to achieve “what we believe to be more sustainable fishing practices” (Quote-19—Table S1). We argue this deployment and distribution of expertise are deeply rooted in conservation practice and participate in hindering stakeholders’ ability to build a common ground upon which to build more equitable management regimes.

## Supplementary Information

Below is the link to the electronic supplementary material.

Supplementary file1 (PDF 397 kb)

## Footnotes

1 The Pacific Community is a regional development, scientific, and technical organization governed by 27 Pacific countries and territories.

2 RESCCUE—Restoration of Ecosystem Services and adaptation to Climate Change (https://resccue.spc.int/).

3 While community members have stressed the significance of the observed ecological effects (increased abundance and size of key fish species), there is debate among marine scientists concerning the ecological effects of Teahupoo’s Rāhui in the absence of baseline data.

4 Members of the *Association Rāhui* considered that the PGEM staff undermined the association by ‘stealing their idea’ of district-level rāhui committees and implementing the fishing committees.
