# Correction: Crafting the success and failure of decentralized marine management

**DOI:** 10.1007/s13280-022-01791-3

**Published:** 2022-09-13

**Authors:** Jean Wencélius, Matthew Lauer, Tamatoa Bambridge

**Affiliations:** 1 Department of Anthropology, San Diego State University, 5500 Campanile Dr., San Diego, CA 92182-6040 USA; 2 PSL Paris University: EPHE-UPVD-CNRS, USR 3278 CRIOBE, BP 1013 Papetoai, 98729 Moorea, French Polynesia

## Correction to: Ambio 10.1007/s13280-022-01763-7

The article “Crafting the success and failure of decentralized marine management”, written by Jean Wencélius, Matthew Lauer and Tamatoa Bambridge, was originally published electronically on the publisher’s internet portal on 25 July 2022 without open access. With the author(s)’ decision to opt for Open Choice the copyright of the article changed on 31 August 2022 to © The Author(s) 2022 and the article is forthwith distributed under a Creative Commons Attribution 4.0 International License, which permits use, sharing, adaptation, distribution and reproduction in any medium or format, as long as you give appropriate credit to the original author(s) and the source, provide a link to the Creative Commons licence, and indicate if changes were made. The images or other third party material in this article are included in the article’s Creative Commons licence, unless indicated otherwise in a credit line to the material. If material is not included in the article’s Creative Commons licence and your intended use is not permitted by statutory regulation or exceeds the permitted use, you will need to obtain permission directly from the copyright holder. To view a copy of this licence, visit http://creativecommons.org/licenses/by/4.0. Open Access funding enabled and organized by the Project ‘A Sea of Connections: Contextualizing Fisheries in the South Pacific Region’ (SOCPacific)(https://socpacific.net/), co-funded by the Agence Nationale de la Recherche (grant number ANR-17-FRAL-0001-01) and the Deutsche Forschungsgemeinschaft (grant number 389654580).

The original article has been corrected.

