# Supplementary material for: Crafting the success and failure of decentralized marine management
Source: Ambio. 2022 Jul 25;51(12):2342–57. doi: 10.1007/s13280-022-01763-7 (PMC9583987; doi:10.1007/s13280-022-01763-7)
Supplement: Supplementary file 1 — Supplementary file1 (PDF 397 kb) [file 13280_2022_1763_MOESM1_ESM.pdf]

**Ambio**

Supplementary Information

**Title:** Crafting the success and failure of decentralized marine management. Moorea, French Polynesia.

*Table S1 – Quotations from meetings or interviews. All names in the quotations have been invented in order to guarantee confidentiality of our informants.*

| Quotation Number | Quotation | Respondent |
|---|---|---|
| 1 | “The number of recreational activities on the lagoon has exploded since 2004 and so have marine traffic and the number of people fishing. We need to be able to manage all these new activities and the conflicts they create between tourist operators, residents and fishers” | PGEM staff member |
| 2 | “Fishers felt excluded from the PGEM, they felt it was designed against them and they distrust the PGEM. This is why, during the revision, we have made a huge effort to secure their participation and implication. It is clear now that the fishers are those which were the most heard during the revision.” | PGEM staff member |
| 3 | “The PGEM is to protect the hotels’beautiful o’iri [Lagoon triggerfish – Rhinecanthus aculeatus]. We have no problem with that, they can have it, it’s no good to eat! But let us catch the other fish.” | Fisher [Afareaitu] |
| 4 | “MPAs which include the nearshore environment are culturally inappropriate. The shore and fringing reef are essential for women’s fishing activities. Men go further out and in high sea. Women use lines, nets and harvest invertebrates on the fringing reef. More importantly it is an essential environment for the upbringing of children. It is through women’s use of the shore that young children are able to connect with and to know their marine environment.” | Cultural activist |
| 5 | “What is PGEM? ‘Plan de gestion de l’espace lagonaire’! That’s France! They have nothing to do in our lagoon and that’s why nobody cares.” | Fisher [Haapiti] |
| 6 | “Let me tell you how it works. Fish follow the rule of God. What you are showing me here is the rule of Humans. Do you think the fish are going to follow your rules? God commands the fish and we fishers are on the lookout of the signs he provides – in the wind, in the color of the flowers on land – to know where to catch the fish.” | Fisher [Afareaitu] |
| 7 | “We shared our knowledge to help the PGEM. We were promised that the protected areas would be Rāhui and fifteen years later they are still the exact same.” | Fisher [Papetoai] |
| 8 | “The French Polynesian government can no longer promote development projects in Moorea in the dark. Having to go through the PGEM committee first, new projects are under the spotlight allowing citizens to know what is going on and they can give their opinion.” | Environmental activist |
| 9 | “My dear companions, you need to rise up. Don’t let yourselves be discouraged. Remember how a handful of us had confronted the anti-riot police in Pihaena in front of the Hilton? […] We have the power to be heard. […] We can fight this new project.” | Steering Committee Member |
| 10 | “At first, it took some time for FP agencies to play ball, but now they almost systematically follow the steering committee’s decisions. After all, Moorea is important politically. It’s the second most populated island and securing votes here is important to the government.” | Environmental activist |
| 11 | “The PGEM is all about money, it is the municipality, the hotels, the tourist-operators who are imposing their rules to the population. The rāhui would come above all of these actors and the committees would make the decisions in each district. We want lagoon management to be carried out by the population and for the population, rather than slicing up the lagoon for different stakeholders.” | Association Rahui Member |

| Quotation Number | Quotation | Respondent |
|---|---|---|
| 12 | "Rāhui, is in the heart of Polynesians. The notion naturally calls for respect. People don't respect the PGEM because it is French. We would simply need to implement a rāhui for people to respect it." | Association Rahui Member |
| 13 | "We first need to get our rāhui set up and running with the approval of the FP government. Only then will we work on bringing mana back into it. You know, our ancestors were able to lift huge stones without even touching them. We've lost this mana and this is why everything is going wrong. With a rāhui, mana will automatically come back and so will respect for nature [use of the Tahitian neologism natura]." | Association Rahui Member |
| 14 | "As a Polynesian, I am myself very attached to the concept of rāhui and I am very happy to see such concepts reemerge. However, I regret that this group has sought to oppose the PGEM instead of collaborating in the design of the revised the PGEM." | DRM staff member |
| 15 | "I wish from all my heart that people, and mostly fishers, could refer to the PGEM as rāhui. Obviously, we are from the old time rāhui which is, in my opinion, no longer possible in Moorea, but it could be considered as a form of modern rāhui. […] It is no longer possible because we are too 'civilized' here in Moorea. There are more and more foreigners living in Moorea. Moreover, there aren't any strong community leaders, recognized by all stakeholders around the island, who could enforce a rāhui." | PGEM staff member |
| 16 | "People think the PGEM is all about the MPAs. Fishers when thy refer to an MPA say 'This zone is PGEM'. What they don't get is that the whole lagoon is PGEM. MPAs are just one of the many tools used by the PGEM." | PGEM staff member |
| 17 | "This system of district-level fishing-committees is quite new for everybody, for the fishers, but for us too. Moorea, right now, is kind of our experimental lab, we are trying out new forms of governance which, we hope, will be transposable to other islands across French Polynesia." | DRM staff member |
| 18 | "This area was picked as an MPA in the first PGEM because fishers had indicated that it was an important fish nursery. […] You need to be thoughtful about future generations, are you really sure you want to go through with this? Who is in favor?"<br>[Authors description:] The question was followed by an awkward silence, participants seemed startled by being given the power to make the final decision. A few fishers hesitantly raised their hands, after what the majority of participants enthusiastically joined in. The DRM agent acknowledged the vote and asked the staff-member taking notes to modify the PowerPoint, thus, codifying fishers' decision into the revision." | DRM staff member |
| 19 | "Well, I have to admit that, in terms of conservation, we are taking a couple steps back. The total area of no-take zones has been significantly reduced. But the whole idea of these committees is to get fishers on board, to have them participate, and to make them realize that we do consider their point of view. We hope that in the end they will realize we aren't their enemy. Once we have them on board and create trustful relations, which is in good course, we will be able to work as a group towards what we believe to be more sustainable fishing practices." | DRM staff member |

| Quotation Number | Quotation | Respondent |
|---|---|---|
| 20 | [When PGEM staff members were asked what constituted a legitimate representative, one staff member stated:] “We seek out people who have multiple functions and who are recognized through different communities. Look at the case of Tutea, he is a respected and knowledgeable fisher, he is a parish deacon and has a strong foothold in the municipality as a councilman.” | PGEM staff member |
| 21 | “We don’t get along with the committee representatives. They are net fishers and they keep harassing us about the fish we catch at night. If we showed up at the meeting I know it would have been a disaster.” | Fisher |
| 22 | “Your [addressing scientists] knowledge is like the titanic and ours like Noah’s arch. Our knowledge seems more rudimentary but which of these ships weathered the storm? […] Our ancestors conquered the Pacific without resorting to inventing the compass, all of this because we have a deep knowledge of our environment and how it works” | Fisher |
| 23 | “The team leading the PGEM pushed us scientists aside. Not only did they not want us to play an active role, but they also simply didn’t want us to take part in the revision.” | CRIOBE scientist |
| 24 | “I don’t understand why the PGEM distrusts us. We aren’t here to harm, the work we are doing here is for the greater good.” | GUMP Station Staff Member |

*Appendix S1 – The fragile balance of a three-way tango*

The final steps of the PGEM-revision as well as recent events have made apparent the fragility of the three-way-tango between the local civil society, the municipality and central authorities. In March 2021, during the last CLEM (see Table 2) meeting during which the final version of the revised-PGEM was presented, the mayor of Moorea asked to modify the zoning of one of the flagship MPAs of Moorea – now labelled as an 'Environmental, user-safety, and sustainable-tourism zone' – in order to enable the project of one of the island's luxury resorts to extend both on-land and over-water bungalows. The extension project, being located at the heart of the protected zone, would have been impossible given the regulations of both the initial PGEM and its revised version. Most CLEM members strongly advised against such a modification arguing that, firstly, it had never been mentioned in any of the 200 revision workshops and public meetings and, secondly, it would further delay the implementation of the revised-PGEM as such a significant modification had to go through a lengthy process of public consultation which could last several months if not years. The CLEM ruled against the proposal and did not include it in the finalized version of the revised-PGEM.

However, several months after the CLEM submitted the revised version of the PGEM to the FP government for final approval, the '*Conseil des Ministres*' (Board of Ministers) – the institutional organ responsible for enacting French Polynesian laws –enacted the final text after unilaterally introducing a zoning mechanism ('*Zone de Développement Prioritaire'* – Development Priority Zone) that could allow the FP government to promote development projects regardless of any regulations imposed by the PGEM. In practice, this was a way to open a window of possibility for the hotel extension to take place without modifying the zoning proposed in the revised version of the PGEM. The decision created an uprising among environmental activists, cultural associations and residents of Moorea among which four organizations filed lawsuits against the FP government on the grounds that the '*Conseil des Ministres*' could not legally enact substantial modifications to the revised version of the PGEM without prior consultation with the CLEM committee who had designed the text.

Local contestation against the government's decision and the hotel's extension project has been particularly strong as the development would occur along one of the island's most popular and scenic beaches. The local uprising crystalized in a large-scale protest held on Temae beach on November 13$^{th}$ 2021. Over 2,000 participants attended the protest which was organized by a recently formed association of residents (seeking to oppose the increasing number of development projects around the island) and by the Protestant Mā'ohi church. The protest took the form of a cultural ceremony during which Polynesian songs, speeches and dances were performed. Each of the island's six parishes of the Mā'ohi protestant church performed in turn and selected some of their members who presented one or several *'orero* (eloquent speech), namely a particular kind called '*pari pari fenua*' which is a form of story-telling reminding the history of a particular place through the enumeration of ancestors, spirits and their links to terrestrial and marine landmarks. The crux of the protest was a ceremony named "*Tahei 'auti*", which can be translated as attaching the *'auti* which is a generic term for several species of the genus *Cordyline*, an important ceremonial plant in pre-contact Tahiti. Participants were asked to bring to the protest a woven rope of *'auti* so that individual ropes would be tied to one another forming a unique rope symbolizing the solidarity of Moorea's population. During the ceremony Hinano Murphy – a prominent community leader and founder of one of the main cultural associations on the Island – publicly presented the ceremony "*as a traditional and solemn ceremony through which all of us can reconnect with one another, reconnect with our land, reconnect with our culture*."

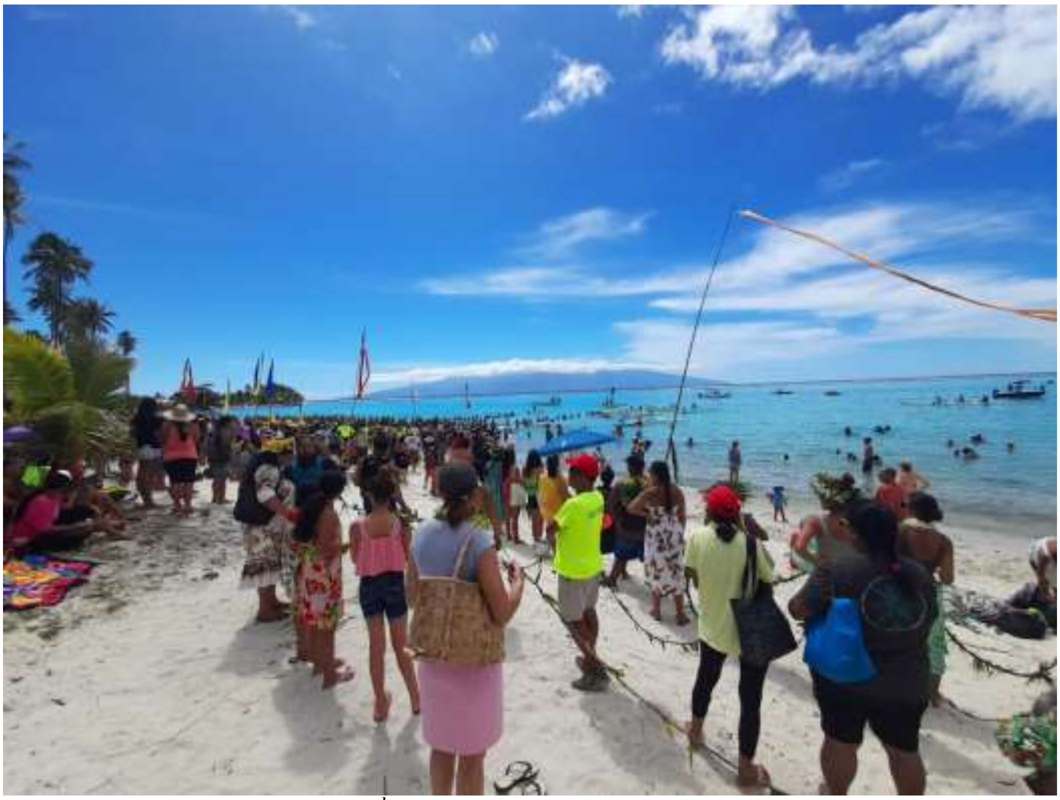

*Temae Beach Protest – Nov 13th 2021 – Tahei 'auti* ceremony (Credits: [masked for peer-review])

In this particular case, the political arm wrestle which occurred around the PGEM revision between the FP government and civil society gave birth to vibrant forms of contestation with activists mobilizing Polynesian concepts and cosmology rather than purely environmental ones. The protest was as much the expression of a willingness to oppose the accelerating development of the island, as the voicing of the need to revitalize Polynesian cultural identity and way of life.
